# The Split Second Effect: The Mechanism of How Equinus Can Damage the Human Foot and Ankle

**DOI:** 10.3389/fsurg.2016.00038

**Published:** 2016-07-27

**Authors:** James Amis

**Affiliations:** ^1^Department of Orthopaedic Surgery, University of Cincinnati, Cincinnati, OH, USA

**Keywords:** equinus, isolated gastrocnemius contracture, plantar fasciitis, insertional Achilles tendinosis, Achilles tendinitis, midfoot arthritis, Charcot arthropathy, biomechanics of gait

## Abstract

We are currently in the process of discovering that many, if not the majority, of the non-traumatic acquired adult foot and ankle problems are caused by a singular etiology: non-neuromuscular equinus or the isolated gastrocnemius contracture. There is no question that this biomechanical association exists and in time much more will be uncovered. There are three basic questions that must be answered: why would our calves tighten as we normally age, how does a tight calf, or equinus, actually cause problems remotely in the foot and ankle, and how do the forces produced by equinus cause so many seemingly unrelated pathologies in the foot and ankle? The purpose of this paper is to address the second question: how does a tight calf mechanically cause problems remotely in the foot and ankle? There has been little evidence in the literature addressing the biomechanical mechanisms by which equinus creates damaging forces upon the foot and ankle, and as a result, a precise, convincing mechanism is still lacking. Thus, the mere concept that equinus has anything to do with foot pathology is generally unknown or disregarded. The split second effect, described here, defines exactly how the silent equinus contracture creates incremental and significant damage and injury to the human foot and ankle resulting in a wide variety of pathological conditions. The split second effect is a dissenting theory based on 30 years of clinical and academic orthopedic foot and ankle experience, keen clinical observation along the way, and review of the developing literature, culminating in examination of many hours of slow motion video of normal and abnormal human gait. To my knowledge, no one has ever described the mechanism in detail this precise.

## Introduction

Foot and ankle problems account for significant percentage of visits to physicians every year, particularly primary care physicians, orthopedic surgeons, and podiatrists. The cost of treating these problems is no doubt an economic strain on all of us (1, 2).

The equinus or isolated gastrocnemius contracture deformity referred to herein is of the non-neuromuscular/non-paralytic type in otherwise healthy individuals. This is why it is often referred to as subtle or silent.

There has been an increasing amount of literature in the last several decades suggesting a link between equinus and many foot and ankle conditions (1, 3–53). Despite this association, little if any evidence concentrating on a mechanism of how the tight calf might actually produce problems in the foot and ankle (3, 4, 11, 15, 18, 31, 32, 44, 54–56).

While we are busy discovering the cause and effect associations between equinus and certain foot and ankle conditions, there has been little interest in determining or explaining how equinus might actually cause damage to the foot and ankle. Two authors have indeed shed light on the cause and effect that equinus exerts on the human foot and ankle. In 1913, Nutt (57) shed considerable light on this subject only to be lost until now. In 2014, Cazeau and Stiglitz (14) described causality to some extent as equinus relates to increased forefoot pressures describing the critical area of the gait cycle as the C-zone when the ankle dorsiflexion is less than 5° and the maximal point of forefoot pressure as the X point and just after.

The incidence of non-neuromuscular equinus in otherwise normal, healthy people (5, 11, 13, 14, 18, 23, 25, 57–59) is not known; however, it is likely far more common than currently perceived. While most would consider a calf that is too tight to be trivial, this could not be further from the truth. The long-term strain produced by equinus, which is usually silent, ultimately leads to progressive, incremental damage in the foot and ankle until it finally becomes symptomatic. Directing treatment toward the most obvious problem, the presenting foot or ankle condition, has traditionally been the main, if not the only, objective; however, emerging data will hopefully point us in a different direction, i.e., the correct direction.

The purpose of this paper is to describe the exact mechanism, the split second effect, by which a seemingly innocuous, subtle equinus contracture causes so much cumulative damage and deformity in the human foot and ankle over time as we age.

The source of the isolated gastrocnemius contracture or equinus has recently been recently addressed by this author (5). The four common categories described in this article: activity changes, physiologic changes in muscles and tendons, genetics, and reverse evolution. An additional fifth source of the acquired isolated gastrocnemius contracture is discussed here in Appendix B.

Finally, considering this knowledge, I assert, if not challenge, that the calf is the singular source of the majority of acquired, non-traumatic adult foot and ankle problems, such as plantar fasciitis, non-traumatic midfoot osteoarthritis, diabetic Charcot arthropathy, insertional Achilles tendinosis, and Achilles tendinitis (Appendix A).

## The Perfect Human Foot

To understand the forces projected upon the human foot and ankle by an isolated gastrocnemius contracture, one must first understand the “perfect foot” (5). The perfect foot, from a mechanical and theoretical standpoint, is the foot that evolution left behind long ago, as humans moved away from being quadrupeds. In understanding the structure of the perfect foot, we are better able to understand, by contrast, the damaging forces equinus places upon the two imperfect feet that we have been entrusted.

Structurally speaking, the equinus foot of a horse or dog is better suited for wear and abuse than the human foot, especially for the long haul. It would be correct to argue that this might be the case because quadrupeds ambulate on four legs distributing the weight on four instead of two feet. However, when considering the foot as a biomechanical structure unto itself and the forces humans put on each of theirs through every day, it is apparent that the construction of the quadruped foot is much more suited for the task required.

**Figure 1:**
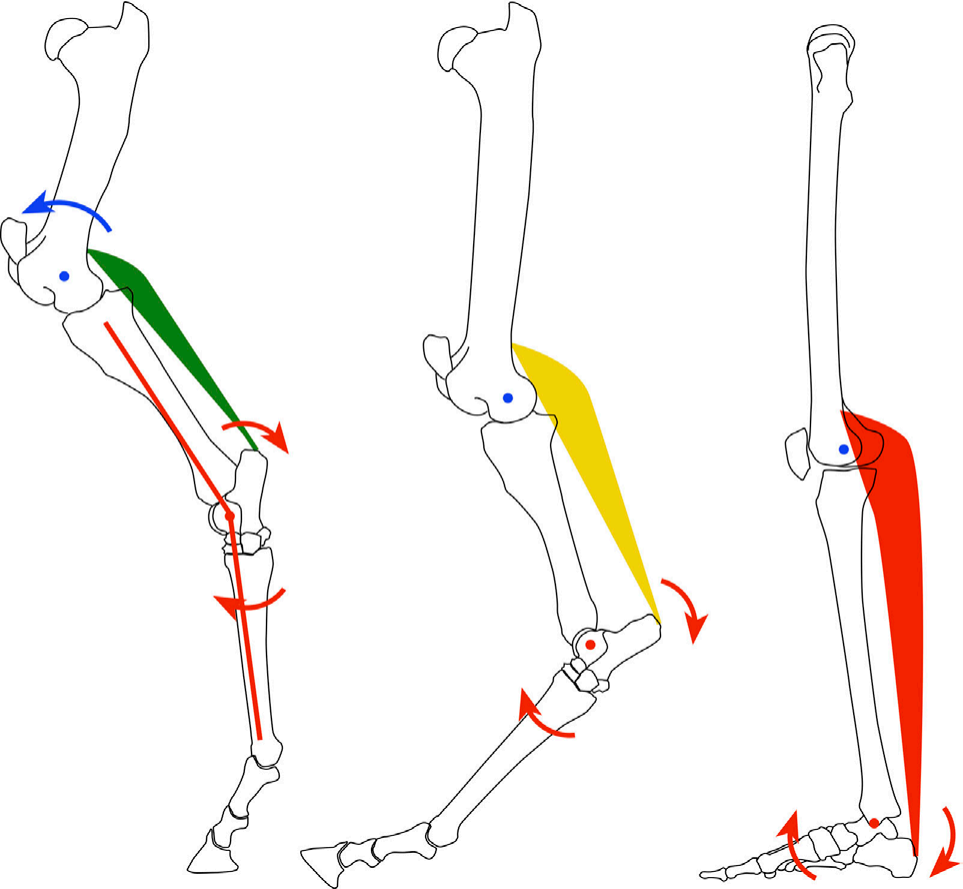
**Diagrammatic representation of the evolution of the equine quadruped (left) to human biped (right)**.

Of course, the weight-bearing platform of an equinus foot is too small for prolonged maintenance of a bipedal gait, considering balance and ground force distribution. So, the human foot wisely evolved and rotated upward through the ankle joint about 70° to permanently place the heel on the ground (Figure 1). This adaptation provided a much larger plantar surface area for improved force distribution, balance, and necessary leverage for bipedal locomotion. However, this new foot position placed the human foot in a biomechanically disadvantaged position due to the leveraged forces placed upon it by ground reaction forces.

With weight bearing, the human foot is oriented such that forces shift from a vertical, mostly compressive load (perfect equinus foot) to leveraged/bending or vector forces resulting in higher compressive (dorsal foot, anterior ankle) and tensile forces (plantar foot, posterior ankle). Understanding the difference between the quadruped, equinus foot and the biped, plantigrade foot is critical to understand the damaging effects of equinus and thus the split second effect.

## Basic Human Gait

Human gait, or the act of bipedal walking, is divided into two basic periods: stance and swing. During the stance phase of gait, the foot is on the ground. The stance phase is divided into five parts: initial contact, loading response, mid-stance, terminal stance, and pre-swing. Furthermore, Perry (60), in her classic book, *Gait Analysis: Normal and Abnormal Function*, looks at the center of stance phase of gait as “progression over the supporting foot,” divided into the heel rocker, the ankle rocker, and the forefoot rocker. For ease of use, the purpose of this theory, and particularly for future discussion applying additional rockers, these rockers will be renamed R1 (heel), R2 (ankle), and R3 (forefoot). The ankle rocker, or R2, is the critical time during gait when the split second effect occurs.

Once the forefoot has fully contacted the ground, R1 is completed and R3 has yet to occur. The body weight is now fully on the stance foot and R2 is engaged as the tibia rotates forward to carry the passenger (body) forward over the ankle joint. In order for humans to progress forward using our unique bipedal gait pattern, the passenger must and will move forward over the planted or stance foot. If the gait is to remain smooth and proceed normally, the calf must have enough flexibility to allow the tibia to adequately rotate over the talus in synchrony with the body above, yet have enough strength to eccentrically control the deceleration at the end of R2. In order for unimpeded completion of R2, the tibia must be allowed to rotate forward upon the ankle or dorsiflex fully. In the normal state, the calf lengthens enough to allow the necessary ankle rotation producing a smooth transition reducing bending vector forces onto the foot and ankle at the end of R2.

## The Split Second Effect

The split second effect is a theory describing how an equinus contracture mechanically damages the human lower leg, ankle, and foot.

There are two types of forces created by the split second effect. *Direct tension* forces are more obvious, occurring at the gastrocnemius muscle, musculotendinous junction, the Achilles tendon, the Achilles insertion at the calcaneus, the calcaneus, and finally the plantar fascia. *Indirect forces* are leveraged upon the foot and ankle and the more esoteric component of this theory.

If the calf becomes too tight, thus limiting its ultimate available length, the rotation of the ankle (R2) is hindered and stopped too early. Yet, the body continues to progress forward over the planted foot, which will produce increased leveraged forces in the foot and ankle. Of course, the ground reaction forces are unavoidable and provide the balance of this equation. The tighter the calf becomes, the higher and earlier the leveraged forces that result.

Early on, these forces generally are not visibly evident as pathologic motion in the foot, and they are undetectable, but the magnified forces are there nonetheless. In other words, the effect is invisible to the casual observer, likely the trained observer, as well as to the person with the tight calves. All the while, the silent, unknown equinus contracture, even in early stages, creates incremental exaggerated leveraged or bending forces upon the tension and compression sides of the foot and ankle. In time, these repetitive forces take their toll, especially as the isolated gastrocnemius contracture increases.

The split second effect describes a critical 120 ms span of time during terminal midstance (R2), just before the heel lifts off the ground, where damaging forces are produced along the whole tension chain, both direct and indirect. These forces are created and magnified by the equinus contracture impeding the forward progress of the tibia or passenger over the ankle. The indirect leveraged forces are exponentially magnified, creating abnormal, pathologic bending or vector stress to the foot and ankle, thus resulting in stress and strain to the joints and supporting ligaments and tendons in a variety of ways ultimately creating an array of non-traumatic acquired foot and ankle pathology (Appendix A).

When we are younger, a normal length gastrocnemius allows the necessary ankle dorsiflexion and smoother near-end-point deceleration as the ankle progresses forward late in R2 just before R3 begins. The ultimate length of the gastrosoleus complex is likely not reached, and the end transition forces are dampened by the compulsory contracture of both the gastrocnemius and soleus (and less end point tension) gradually producing a softer end point. There is room to spare when it comes to the amount of gastrocsoleus complex length that is necessary. This creates an easier, more cushioned force transfer upon the foot and ankle below. However, even when the calf is supple and the ankle has “adequate” dorsiflexion, there is still a transfer of progressive leveraged stress to the foot and ankle even under the most “normal” of conditions. This is the burden we as humans must bear in order to have a bipedal gait.

The split second effect is divided into two separate mechanical components: ankle dorsiflexion and knee extension.

## Ankle Dorsiflexion Component

**Figure 2:**
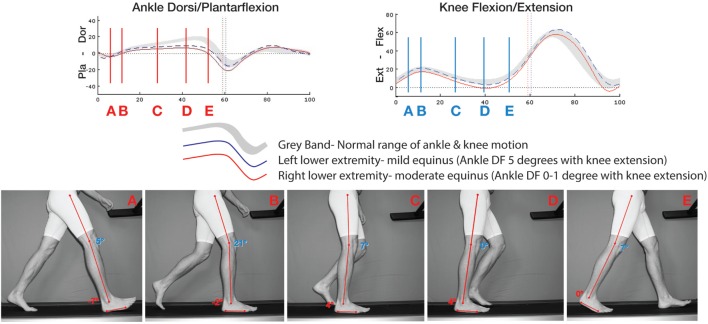
**Motion analysis compared to same subject on treadmill shot at 250 FPS of subject with bilateral equinus and history right plantar fasciitis who is asymptomatic at time of testing**. At 28–45%, frames C–D depict the timing of the split second effect. There is accentuated knee extension and a failure of ankle dorsiflexion. This lack of normal ankle dorsiflexion motion gives rise to leveraged forces upon the foot and ankle.

The last half of midstance, R2, is exactly where the split second effect occurs. It begins when the swing phase foot starts to pass by the stance phase foot and ends just as the stance heel lifts from the ground to the earliest part of R3. For most of us, walking at a normal pace, this usually lasts about 120 ms, or just over 1/10th of a second. This timing was derived from video at 250 frames per second (FPS) and limited motion analysis. Figure 2 is a 57-year-old individual with a history of plantar fasciitis on the right and no history of foot or ankle problems on the left. At test time, the plantar fasciitis is asymptomatic. Frame C to D is when the split second effect occurs and ankle dorsiflexion fails to progress. This occurs at approximately 28–45% of the gait cycle.

With equinus, the leveraged forces transmitted to the foot and ankle during this split second become exponentially worse as the gastrocnemius prematurely reaches its endpoint. The tighter the calf, the sooner and more abrupt the endpoint is reached. This in turn makes the foot and ankle directed forces higher, peak faster, and last longer. These forces can of course be mitigated by the characteristic compensatory “start up” gait alteration such as seen in plantar fasciitis and many other related problems. The start-up limp creates the characteristic gait that compensates for an even tighter gastrocnemius created by rest and lack of calf tension created by the Law of Davis (57). Just as the higher tension is reached, R2 is not completed, and the tibia does not complete its rotation over the talus. It is only after this temporary additional contracture is slightly re-lengthened by walking does the gait return to “normal,” at least as perceived by the patient.

Over time, and as a result of taking thousands of steps per day, the foot and ankle succumb to an occult, unrecognized “overuse of imbalance,” which ultimately leads to damage. This damage can create defined pathology, such as plantar fasciitis, midfoot arthritis, insertional Achilles tendonosis (Haglund’s deformity), and posterior tibialis tendon dysfunction, to name just a few (appendix A).

## Knee Extension Component

In the course of looking through hours of slow motion video of many different people walking, both normal and pathologic, a second consistent component critical to the split second effect was noticed. It was observed that, at the exact same time, the ankle was prematurely ceasing to rotate due to the isolated gastrocnemius contracture, the ipsilateral knee abruptly comes to terminal extension or straightens out fully. This might seem unimportant until one considers that the gastrocnemius crosses three joints: the subtalar joint (negligible in this model except for posterior tibialis dysfunction), the ankle, and the knee. With this simultaneous knee extension, the gastrocnemius is pulled even tighter at *just the wrong time*, when the ankle has ceased to progress forward and ankle dorsiflexion has stopped. This knee extension produces two perfectly timed and simultaneous opposing forces, both of which only increases the direct tension force and indirect leveraged force placed on the foot and ankle below, by causing a further abrupt tension.

In the case of where equinus is present, there is likely an associated increased knee extension or even knee hyperextension occurs secondary to the restricted ankle dorsiflexion. Note in Figure 2 how both knees extend outside the normal gray band and the right (plantar fasciitis side) knee extends fully to 0°.

To be perfectly clear, the ankle dorsiflexion or R2 is distally halted by the calf contracture too early, while the knee abruptly straightens to full extension pulling a now inflexible, tensioned calf even tighter over a very short period of time. The result is the tension in the calf exponentially rises over a split second creating indirect damaging compressive and tensile forces onto the foot and ankle, direct tensile forces along the posterior/plantar tension chain, and likely the knee as well. It would be like two people with the same rope tied around each other’s waists running away from each other in opposite directions until they reach the end of the line. Twice the force in half the time: a split second.

**Figure 3:**
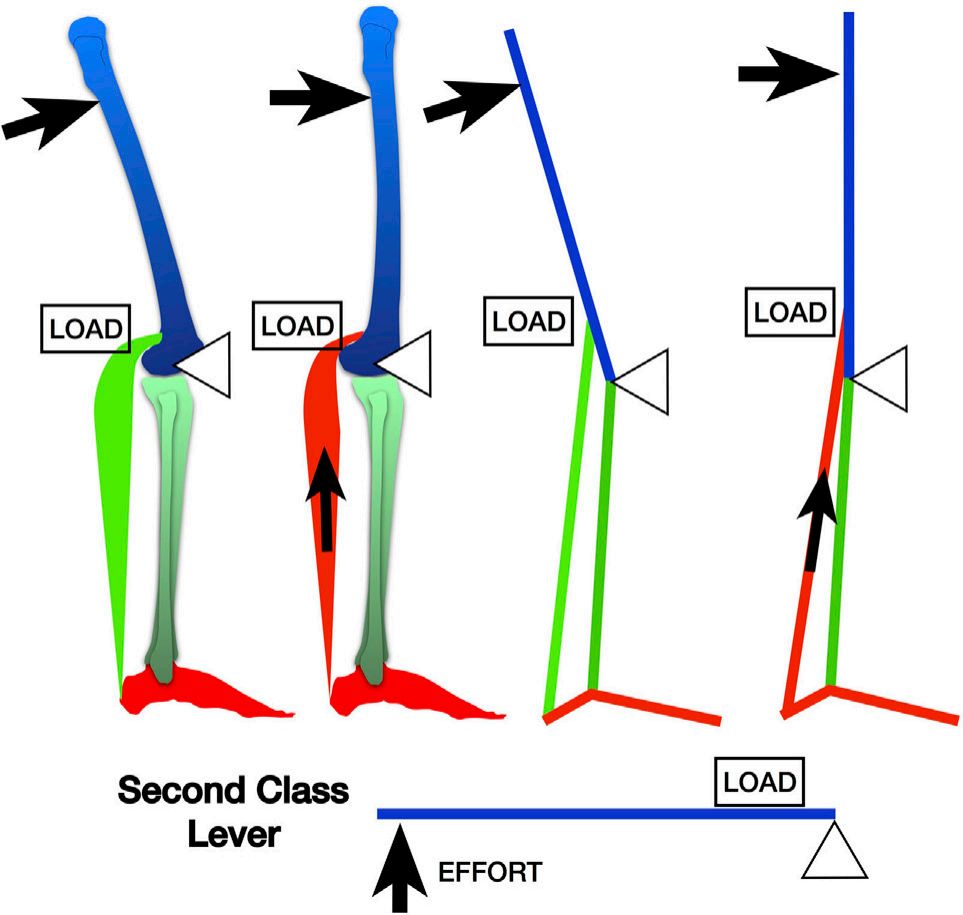
**The leveraged forces produced from terminal knee extension during the split second effect via the gastrocnemius by a second class lever mechanism**.

There is a second damaging mechanical force produced by the knee extending at this time adding to the amount of tension produced. The short arc Class II lever at the extending knee (fulcrum) produces high tensile forces through the calf because the fulcrum is short (distance from axis of knee motion to insertion of gastrocnemius) compared to the long lever of the femur (the lever). This is the finishing blow, so to speak, producing a very high tensile, leveraged force over a very short time (Figure 3).

## Clinical Example: The Split Second Effect and Midfoot Strain

Examples of direct tension forces are simple and easy to comprehend. Too much tension produced by the split second effect anywhere along the posterior/plantar tension chain can cause damage producing the likes of insertional Achilles tendinosis or plantar fasciitis, for example.

**Figure 4:**
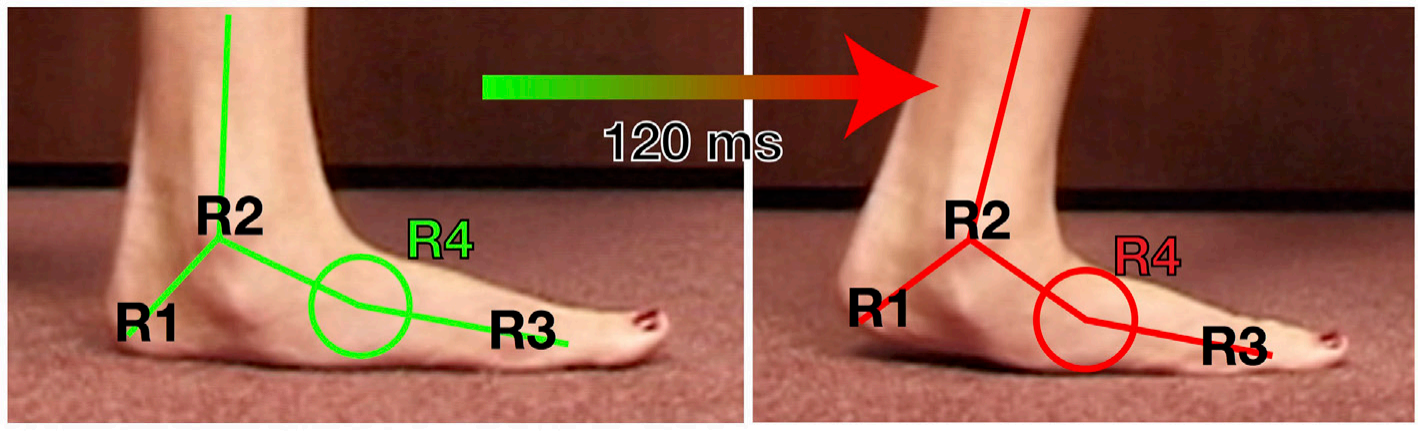
**Video still capture from video shot at 250 FPS of a 23-year-old asymptomatic female with painless flexible flatfeet**. This visibly exhibits the motion transfer from the ankle (R2) to the midfoot/transverse tarsal joint (R4) when there is equinus. If this flexibility at R4 seen here visibly is not present, such as a normal, less flexible midfoot, the motion is replaced by leveraged forces to the midfoot joints or to the posterior tibialis tendon/spring ligament at the transverse tarsal joint.

As a clinical example of the more elusive indirect forces produced, one of the many areas of stress placed onto the foot due to the split second effect is at the midfoot, defined here as R4. Because the ankle rotation (R2) is reduced or stopped, either the gait adapts with early heel raise or the foot starts to create a “new ankle” or point of rotation, R4. In a normal foot setting, the stresses thrust upon the midfoot cannot actually be seen *per se*; however, this abnormal motion is well illustrated in this asymptomatic 23-year-old with congenital flexible flatfeet (Figure 4).

Two very common problems we see, non-traumatic midfoot arthritis (4, 9, 13, 18, 61) and Brodsky type 1 and 2 Charcot arthropathy (4, 13, 18, 20, 29, 42), do not just appear by magic. There is no doubt that both of these very common deformities result in part, if not completely from inevitable calf contractures, mediated by the split second effect and R4 bending forces through the midfoot (Figure 4).

An equinus contracture, with associated hamstring contractures (12) and the split second effect could possibly have implications upon the knee and idiopathic knee osteoarthritis. Additionally, this all compounds up the chain right to the hips and back. However, the purpose here is to look at the effect on the foot and ankle.

## Discussion

Evidence of the association between the isolated gastrocnemius contracture or equinus and the majority of non-traumatic acquired foot and ankle pathology abounds (1, 3–53). We are awakening to a new era of understanding the mechanics and function of the human foot and ankle. There is a simple, singular, usually silent, and remote cause for the majority of non-traumatic acquired foot and ankle pathology, and mechanically, it creates cumulative damage to the foot and ankle through leveraged forces. In short and in this author’s opinion, equinus is the primary mechanical common denominator that leads to the majority of acquired non-traumatic foot and ankle problems by indirect leveraged means as well as direct forces along the posterior/plantar chain. There can be no more room for the standard thinking that these resultant foot and ankle problems arise just because we are getting older or we are obese or they are just random, or that an equinus contracture is only a part of the equation. Equinus is the equation.

These non-traumatic acquired foot and ankle problems are mechanical in nature, an “overuse syndrome of mechanical imbalance” of sorts, and the end result is a chronic inflammatory process. As a result, traditional non-operative treatment generally fails because it is misdirected at the obvious problem, the foot and ankle inflammatory issue. Treating the resulting inflammatory process can no doubt temporarily alleviate symptoms; however, it is still at best palliative and fails to treat the underlying cause. No wonder mentions and anecdotal evidence pointing out the failure of conservative treatment of these resulting problems [Appendix A] dominates in our literature (11, 21, 23, 62–64).

Why is there such a resistance to the possibility that an equinus contracture is central to so many foot and ankle problems as we age? Much of our literature can certainly be interpreted as doubtful if not ignoring it altogether. Most importantly, the methods of foot and ankle care and treatment probably unlike any other sector of medicine are fueled by urban myth, conflicting research, quick fix society, and a medical industry concerned with palliative-directed treatment.

The concept of the subtle, non-neuromuscular acquired equinus contracture is not new. Nutt (57) described “Shaffer’s foot” over 100 years ago. He stated, “…this condition is a shortening of the gastrocnemius and of the soleus. This shortening is not enough to produce the deformity of equinous but limits [ankle] dorsal flexion.” In this forgotten, but landmark 1913 book *Diseases and Deformities of the Foot*, Nutt described the abnormal stresses place upon the foot by the contracted gastrocnemius.

In 1995, Hill (23) stated “equinus reflects ‘acquired deformity’ related to factors of lifestyle that leave the posterior muscles at a physiologic disadvantage toward maintaining flexibility.” Furthermore, he went on to state, “Gastrosoleal stretching is an important treatment modality that can lead to a higher success rate of conservative treatment. It can decrease the need for foot surgery, and significantly reduce the number of failed or serial surgical procedures.”

In 2002, DiGiovanni et al. (18) coined the term “isolated gastrocnemius contracture” and rekindled the idea of the gastrocnemius producing foot and ankle pathology with out any proposed mechanism. The sheer importance of this seminal article has been mostly overlooked by the great majority of the orthopedic and podiatric community, even today. They stated, “Our experience has suggested that the gastrocnemius muscle in particular is the predominant deforming force in people with structural breakdown or chronic pathological changes related to the foot and ankle. We suspect that contracture of this muscle is not only common but often partially responsible for many of the chronic forefoot and midfoot symptoms identified in non-neurologically impaired patients.”

I would disagree with two critical assumptions they made. First, I would disagree with their comment “We suspect that contracture of this muscle is not only common but *often partially responsible* for many of the chronic forefoot and midfoot symptoms identified in non-neurologically impaired patients.” I believe that the contribution of the isolated gastrocnemius contracture is the singular cause of the many associated pathological conditions described in the literature, and is partially responsible much less often. These authors also stated, “We further postulated that the difference would be present whether the knee was extended or flexed.” It is my hypothesis that the previously unknown simultaneous extension of the knee is critical to the synergistic damage delivered to the foot and ankle by the isolated gastrocnemius contracture and is described here as the split second effect.

In 2002, Porter et al. (56) reported on isolated calf stretching for plantar fasciitis demonstrating a measurable and excellent improvement in ankle dorsiflexion and pain relief. The dorsiflexion gained over 4 months went from average ankle dorsiflexion of 5.1–13.5°. Foot and ankle scores (pain relief) improved on average 56–90%. They stated “The increase in flexibility correlated with a decrease in foot and ankle pain, and an increase in foot and ankle function. This correlation should be emphasized to physicians and patients alike.”

Cazeau and Stiglitz (14) provided additional insight into this biomechanical aberration describing the mechanism of equinus leading to increased forces placed upon the forefoot. They termed this timing of forefoot overload resulting from equinus as the “C-zone,” defining it as “the lack of EMG activity points out the passive nature of gastrocnemius stretching.” They also described the “X point” in the middle of the C-zone as the point the gastrocnemius reaches its maximal stretching and just after. While no force plate analysis was provided, it can be safely assumed that the forefoot pressures increase significantly at the X point. Their work endorsed addressing the calf, but by surgical lengthening and characteristically denounced calf stretching as minimally effective stating, “various clinical presentations and pathologies are described in which stretching as a single treatment is probably insufficient.” While more work appears to be necessary, the evidence would disagree with the ongoing inference that calf stretching is generally ineffective (12, 23, 56, 59, 65).

In the last two decades, the evidence has been mounting supporting that the gastrocnemius is associated with many foot and ankle problems, but little, if any, reason as to the cause and effect has been offered until now. The split second effect concept is like a key to open the lock few even knew existed; here is the problem explained simply. No doubt many more studies will and should emerge to both qualify and quantify equinus and its role on the demise of the human foot and ankle. It is likely that high resolution, ultra high-speed gait lab motion analysis coupled with force plate analysis should and will show this theory to be correct, as well as provide more defined details.

Misperception and conventional wisdom has pervaded our literature in regard to etiology of the majority of non-traumatic acquired foot and ankle pathology. Much of the current thought and evidence on the epidemiology of these non-traumatic acquired foot and ankle pathologies is by association and subjective (3, 29, 33, 44, 55). The generally accepted factors cited in the literature and on the internet, such as obesity, sedentary life style, medical co-morbidities, shoe wear, concrete floors, overuse, etc., likely have a common pathway to these associated foot and ankle problems via an equinus contracture. In other words, these factors, while they may indeed be commonly present, contribute minimally if at all in a direct manner to the resulting problems or pathology in the foot and ankle. Each of these factors at best creates an additional accelerated avenue to equinus, which in turn causes the foot and ankle pathology by way of the split second effect.

For instance, it is more or less thought by many that the acquired flatfoot deformity is somehow a random, unknown problem or due to obesity and that calf contractures might come about secondarily. In this scenario, acquired equinus might at most be considered to only contribute to an acquired flatfoot deformity usually after the fact (22). The studies done commenting on the epidemiology of this problem, as well as many other foot problems, point to other collateral or associated factors noted above and that is where they stop. The calf contracture indeed worsens as the flatfoot deformity progresses via vicious cycle, but it is still likely the inciting underlying cause.

The singular and real cause of these problems is a contracture of the gastrocnemius muscle, which is camouflaged in this large list of collateral, minimally contributory risk factors. Every other factor cited as a cause of foot and ankle problems is mediated by contributing to the degree and/or rate of an already contracting human gastrocnemius (5). In other words, these co-morbidities promote gastrocnemius tightness, which in time causes incremental damage to the foot and/or ankle. This concept cannot be overstated or we will continue to aim our treatment efforts at the wrong target.

To put it very frankly, obese people have calf lengthening and resolve their recalcitrant Achilles tendinosis and yet they are still overweight. Employees who work on concrete have a calf lengthening procedure, resolve their midfoot arthritis pain, and return to work pain free on the concrete. A runner with plantar fasciitis definitively stretches their calves and moves on, pain free back to running.

There are still questions that must be answered. Is calf stretching effective? The evidence seems to support it, yet as a definitive treatment, it is very poorly embraced and the current momentum in this area is unfortunately toward surgical lengthening. Poor compliance with calf stretching would certainly lead to failure as seen in so many areas of medical care (66–68). More importantly, can gastrocnemius stretching be preventative? While there is little if any evidence to support this, further work in this area would be prudent.

Certainly, not everyone will develop significant damage producing isolated gastrocnemius contracture in their lifetime. What is the incidence of equinus? And who is prone to develop it and can we identify them and apply preventative stretching before pathology develops? Are there recognizable subtle gait variances that produce equinus?

Certainly, in most cases, equinus develops bilaterally and approximately equally. Then why are resulting problems often present on just one side or the other? One very tangible and very common cause of unilateral equinus is one limb that is left fallow for a period of time due to injury, or elective limb surgery, or recovery from a total knee only to develop plantar fasciitis in the ensuing months.

Given that we as a culture finally embrace subtle equinus as a significant problem, should we stretch calves or have them surgically lengthened? What guidelines for stretching versus surgically lengthening might there be developed? The current guidelines for considering surgery after 6 months of failed non-operative treatment is likely incorrect. The 6-month time frame is adequate; however, what defines the failed non-operative treatment? Traditionally speaking as well as today, non-operative treatment for these issues includes a random inconsistent mixture of palliative conservative measures directed at the obvious Achilles, foot or ankle problem and does not address the equinus (23). Certainly, the current trend is toward surgical correction of which this author disagrees.

## Conclusion

It appears that merely being human places us at risk of developing acquired non-traumatic foot and ankle problems. This damage is mediated through our unique anatomy and the gastrocnemius that tightens for a number of reasons (5). The split second effect explains exactly how a silent equinus contracture, that seemingly has little to do with the foot and ankle below, can gradually cause significant harm when left undetected and unattended.

The isolated gastrocnemius contracture must be addressed as the definitive treatment for many if not the majority of non-traumatic acquired foot and ankle pathology (Appendix A). While treating the obvious foot or ankle problem is advisable and can be of great benefit to the patient, it must be considered only as adjunctive and palliative.

This crucial concept must be vetted and passed on to as many people as possible and as fast as possible. We have already waited over a century since first described by Nutt (57); let us not wait too much longer. Better yet, going forward, we must promote prevention in the form of proactive daily calf stretching before the damage occurs.

## Author Contributions

This article in its entirety considering originality, views, concept form, authorship and future communication is the sole responsibility of JA, MD.

## Conflict of Interest Statement

The author declares a potential conflict of interest with respect to the publication of this article and the One Stretch^®^, but not to this original research or authorship. The reviewer, KO, and handling Editor declared their shared affiliation, and the handling Editor states that the process nevertheless met the standards of a fair and objective review.

## Appendix A

List of non-traumatic acquired foot and ankle pathology associated with an equinus contracture is described below.

### Compression/Dorsal/Anterior

Generalized foot pain especially associated with start up pain or stiffness
Shin splints/Medial tibial stress syndrome
Midfoot osteoarthritis
Jones/Fifth MT metadiaphyseal stress fracture
Ankle arthritis/Anterior ankle spurs (prevention)
Navicular stress fracture
Metatarsal stress fractures (prevention)
Sinus tarsi syndrome
Diabetic Charcot arthropathy

### Tension/Plantar/Posterior

Plantar fasciitis
Sever’s disease
Posterior Tibialis Tendon Rupture (PTTR)-Acquired flatfoot deformity
Insertional Achilles tendinosis/Haglund’s deformity
Achilles tendinitis/tendinosis
Achilles tendon ruptures (prevention)
Recurrent musculotendinous Achilles ruptures
Calf cramps at night/Charley horse
Second MTP synovitis/plantar plate rupture which leads to hammer toe
Morton’s neuroma
Diabetic malperforans forefoot ulcer formation
Hallux valgus/Bunion
Hallux rigidus

## Appendix B

The rationale as to why calf contractures develop in humans as we age was addressed (5) with one omission: adolescents. I will take this opportunity to address and add this *fifth* category for the shortened gastrocnemius: *bone/muscle growth mismatch*. In this category, the gastrocnemius may well be “normal” length genetically speaking, but the tibia is too long creating a relative or situational isolated gastrocnemius contracture or equinus.

This mismatch is found in the adolescents with Sever’s disease (50, 51, 65, 69). It is no coincidence that Sever’s disease occurs just after or late in the physiologic growth spurt most adolescents go through. In this case, the longitudinal growth of the tibia and fibula temporarily outpaces the gastrocnemius and one gets a “situational” isolated gastrocnemius contracture. The innocent calcaneal apophysis is pulled from both ends being placed under excessive tension placing the split second effect into action. It is the pediatric form of plantar fasciitis, nothing more, nothing less.

I believe in time we will find in another subset of this category that excessively tall people also have an increase in isolated gastrocnemius contracture-generated foot and ankle pathologies due to this same mismatch. In this case, the skeletal growth end point is too long for the gastrocnemius muscle, which is too short. While there are no published comparison statistics this author can find, all one has to do is notice the preponderance of foot and ankle problems, particularly plantar fasciitis, encountered in the NBA compared to other sports.
